# Community Outreach and Engagement in Cancer Research Through a Biobank Clinic at Shaukat Khanum Memorial Cancer Hospital and Research Centre, Pakistan

**DOI:** 10.7759/cureus.55179

**Published:** 2024-02-28

**Authors:** Asim Farooq, Muhammad Hassan, Asif Loya, Kashif Asghar

**Affiliations:** 1 Basic Sciences Research, Shaukat Khanum Memorial Cancer Hospital and Research Centre, Lahore, PAK; 2 Pathology, Shaukat Khanum Memorial Cancer Hospital and Research Centre, Lahore, PAK

**Keywords:** shaukat khanum memorial cancer hospital and research centre, biobank clinic, biobank, cancer research, community outreach and engagement

## Abstract

Introduction: Cancer's increasing prevalence across the globe emphasizes the urgency for continued research, prevention, and accessible healthcare to mitigate its impact on individuals and communities. While there have been significant advances made towards controlling cancer morbidity and mortality in recent decades, Pakistan continues to experience a markedly elevated burden of the disease. With this study, we aim to raise awareness about biobank research within the cancer patient community, fostering participation and collaboration to advance the fight against cancer through vital research contributions.

Methods: In October 2022, we initiated the biobank clinic at Shaukat Khanum Memorial Cancer Hospital and Research Centre (SKMCH&RC). Here, patients underwent screening and received invitations to voluntarily participate in biobank research. During these interactions, we engaged patients in discussions about the significance of biobank research, addressed their concerns, and encouraged their participation in advancing our research endeavors. Two-sample independent t-tests were performed to compare the mean number of participants in pre-clinic and post-clinic cohorts.

Results: This research involved a total of 958 participants, with 312 participants enrolled before the clinic and 646 participants enrolled after the clinic. We have observed a noticeable increase in the participation of cancer patients in our research endeavors since the inception of the biobank clinic (p-value<0.001). Over an 11-month time frame, we scheduled appointments for 759 patients, and out of those, 656 patients availed themselves to visit the clinic. Impressively, we achieved the enrollment of 646 patients into the clinic, reflecting an exceptional consent rate of 98.47% for their active involvement in our research initiatives. This underscores our commitment to conducting comprehensive discussions and providing thorough explanations regarding the ethical and procedural aspects of our research.

Conclusion: Biobank clinic plays a pivotal role in raising cancer awareness and fostering research participation, especially in regions with limited healthcare infrastructure and lower literacy rates. It emerges as a community-engagement model that aligns research with local needs, ensuring its relevance and benefit to the population.

## Introduction

Cancer is a global health concern and a prominent contributor to worldwide mortality [1]. According to the GLOBOCAN 2022 report, there were 19,965,054 reported cases of cancer, resulting in 9,736,520 deaths globally [2]. Notably, Pakistan's population accounts for 2.99% of the global population, securing its position as the fifth most populous country in the world [3]. According to the GLOBOCAN 2022 report, there were 185,748 reported cases of cancer (including non-melanoma skin cancer), resulting in 118,631 deaths [4]. In light of these figures, cancer research becomes imperative and it is essential to advance our knowledge, improve outcomes for cancer patients, and ultimately work towards a world where cancer is more preventable, treatable, and manageable.

Compared to the global strides in healthcare and research, low- and middle-income countries (LMICs), including Pakistan, encounter several substantial challenges [5, 6]. These encompass limited financial and infrastructural resources, inadequate healthcare access, deficiencies in health information systems and infrastructure, and a heavy disease burden [5, 6]. Moreover, LMICs contend with inconsistent and cumbersome regulatory processes that often hinder advancements in both healthcare and research. Furthermore, the low literacy rate within Pakistan’s population emerges as a significant barrier to research participation, as it limits individuals' ability to comprehend the research process and make informed decisions [7]. This is compounded by a lack of knowledge about how research works and can lead to misconceptions about the purpose and potential benefits of research studies. This, in turn, contributes to therapeutic misconceptions, where participants erroneously believe they will receive immediate therapeutic benefits from research participation. Addressing these multifaceted challenges through education and improved communication is essential to ensure ethical and informed research participation.

Amidst these formidable challenges, Shaukat Khanum Memorial Cancer Hospital and Research Centre (SKMCH&RC) stands as a beacon of hope for cancer patients in Pakistan. The hospital's mission is to deliver the highest standard of care while actively engaging in research aimed at advancing cancer treatment and understanding its underlying causes. In line with the commitment to promote cancer research, SKMCH&RC established a biobank in 2019 [8], which currently contains 1158 fresh frozen samples from 17 distinct tumor sites [9], accompanied by comprehensive demographic and clinicobiological data. Recognizing the paramount importance of raising awareness about the biobank and research among the cancer patient community, SKMCH&RC took a significant step by establishing a biobank clinic in 2022, addressing this crucial need and fostering a collaborative environment for advancing cancer research in Pakistan.

## Materials and methods

Patient selection

The process of involving treatment-naïve cancer patients who are scheduled to undergo surgical resection in our biobank initiative is a well-structured and patient-centric approach. We begin by subjecting these patients to meticulous scrutiny through multidisciplinary cancer conferences. Patients aged 18 years and older, and confirmed to have a malignant tumor, are eligible for enrollment in the biobank. Exclusion criteria apply to patients who have undergone neoadjuvant chemotherapy, those with an active infection, or individuals with a tumor size less than 10mm.

Patient consenting and enrolment

Once a patient is deemed eligible and expresses their willingness to participate, they are scheduled for a dedicated biobank clinic appointment. It is important to emphasize that patient participation in the biobank is entirely voluntary, and they are provided with detailed information regarding the rationale behind the biobank, the procedures involved, as well as the potential risks and benefits associated with contributing to this research initiative. Our healthcare professionals engage in comprehensive discussions with the patients, ensuring that they fully understand the implications of their involvement.

Prior to any sample collection, a critical step in the process involves obtaining informed consent from the cancer patients. The biobank coordinator, who is well-versed in good clinical practices, follows international ethical guidelines during this phase. The informed consent form is thoroughly explained to the patient, and sample collection is initiated only after receiving written consent. Importantly, we emphasize to our patients that they have the prerogative to withdraw their consent at any point in time, and in such cases, any collected samples are promptly and securely destroyed.

Protection of patient data confidentiality

To uphold the highest standards of patient data confidentiality and security, we implement stringent measures. Instead of using patient names or personal identifiers, a unique code is assigned to each sample, ensuring that patient information remains completely anonymous. Access to the patient database is strictly limited to authorized personnel, ensuring that patient privacy is upheld at all times. Additionally, all paper documents are securely stored under lock and key, while computerized records are protected with robust security codes, further safeguarding the integrity and privacy of patient data. Our commitment to ethical research practices and the protection of our patients' rights and information remains unwavering. The establishment of the biobank received official approval from the Institutional Review Board (IRB) of SKMCH&RC (IRB-16-09), underscoring its ethical foundation and commitment to research integrity. Our informed consent procedures adhere rigorously to the ethical principles outlined in the Declaration of Helsinki, ensuring the utmost respect for the rights and well-being of research participants.

In this ongoing investigation, we have systematically assessed the efficacy of the biobank clinic as an instrument for fostering community engagement within the framework of cancer research at SKMCH&RC. The study was conducted over a duration of 21 months, commencing in January 2022 and concluding in September 2023.

Biobank services

Our biobank is integral to advancing medical research through a comprehensive range of services. This includes meticulous collection, processing, and standardized long-term storage of various tissue specimens. Its efficient data management ensures secure handling of participant information, while prioritized quality control measures guarantee sample integrity. The biobank facilitates researcher access to stored samples and data, fostering collaboration and scientific advancements. Offering collaborative research support and adhering to ethical standards, our biobank tracks patient outcomes, providing valuable longitudinal data.

Statistical analysis

To assess the normality of both groups, namely the pre-clinic and post-clinic cohorts, we employed a quantile-quantile (QQ) plot. Hence, a two-sample independent t-test was conducted to compare the mean number of participants between the two groups. Additionally, in our data exploration, we used the capabilities of R programming (R Foundation for Statistical Computing, Vienna, Austria) to craft spatial maps for the purpose of visualizing geographical data. For analysis of differences in age, gender, region, and cancer types between both the groups, data were entered and analyzed using SPSS software, v.25.0 (IBM Corp., Armonk, NY). Quantitative variables like age were calculated by mean and standard deviation. Qualitative variables like gender and region were calculated as frequency and percentage. The chi-square test was used to compare the categorical data, and an independent t-test was used to compare the continuous variable concerning the categorical variable if data were normally distributed after checking the normality of the data; otherwise, the Mann-Whitney U test was applied. A p-value of ≤ 0.05 was considered significant.

## Results

**Figure 1:**
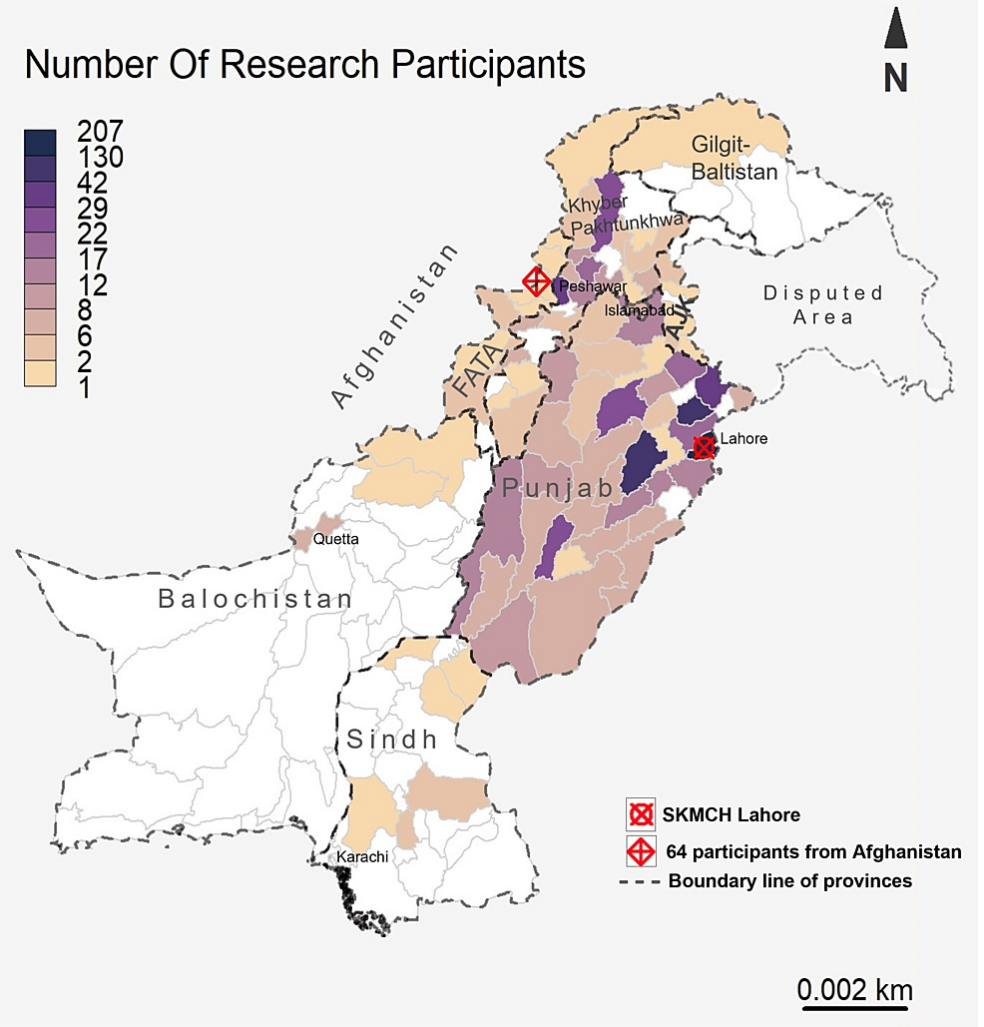
Geographical representation of cancer patient research participants (n=958) across Pakistan (image credits: Muhammad Hassan)

Our community outreach and engagement endeavors for cancer research involve patients hailing from diverse regions across the country, as illustrated in Figure 1. We used R programming to construct the spatial map, facilitating the visualization of geographical patterns and trends associated with the participants in different regions of Pakistan. These regions encompass Punjab, Khyber Pakhtunkhwa, Sindh, Balochistan, Azad Jammu and Kashmir, Gilgit Baltistan, and the Federally Administered Tribal Areas (FATA). Notably, our research initiatives also extend to include patients from Afghanistan who are receiving treatment at our hospital.

**Figure 2:**
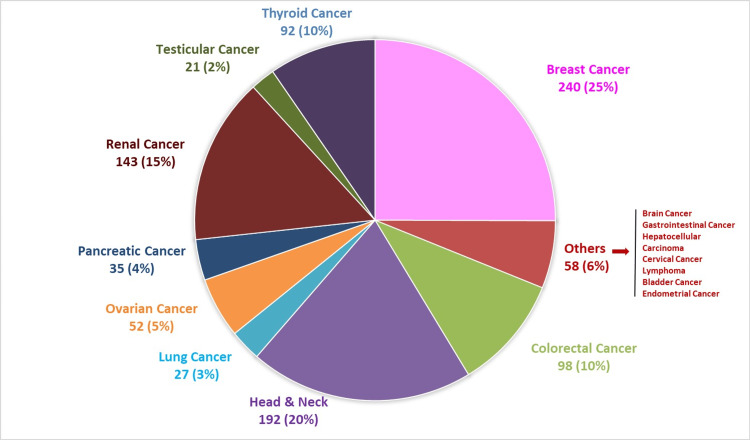
Breakdown of patients enrolled in the biobank clinic by cancer type (n=958)

Currently, we are in the process of enrolling patients from a diverse range of 17 distinct cancer sites. The distribution of the patients, categorized by their specific cancer sites, is shown in Figure 2. Among these, the preeminent share of the cancer population of Pakistan is attributed to individuals diagnosed with breast cancer (n=240; 25%), head and neck cancer (n=192; 20%), renal cancer (n=143; 15%), colorectal cancer (n=98; 10%) and thyroid cancer (n=92; 10%). 

The inception of our biobank clinic in November 2022 marked a pivotal moment in our research approach. Before the inception of the clinic, patients were required to visit the research office, which was situated independently of the oncology clinics within the hospital premises. The physical separation between the research office and the oncology clinics created barriers to accessibility, potentially deterring patients from actively participating in research. To address this issue and enhance patient engagement, we introduced the biobank clinic adjacent to the oncology clinics in the hospital's outpatient department (OPD). This strategic relocation significantly streamlined and organized patient access to our research opportunities.

The dataset encompasses 958 participants, with a mean age of 47 ± 13.3 years, revealing a varied demographic. Gender distribution indicates 40.8% males and 59.2% females, suggesting a higher representation of women. Region-wise, the majority hails from Punjab (68.6%), followed by Khyber Pakhtunkhwa (18.4%), and Afghanistan (6.7%). The dataset comprises individuals with diverse cancer types, with breast cancer being the most prevalent (25%), followed by renal cancer (15%) and head and neck cancers (20%) as shown in Table 1. Furthermore, the comparative analysis between the pre-clinic and post-clinic groups revealed statistically significant differences in both gender distribution (p = 0.04) and the prevalence of various cancer types (p = 0.001) (Table 1).

**Table 1:** Demographic and cancer profile of research participants A p-value of <0.05 was considered significant.

Characters	Total number (%) 958 (100)	Pre-clinic	Post-clinic	p-value
Age (years)				0.27
Mean	47±13.3	47.8±14.1	46.9±12.86	
Gender				0.04
Male	391 (40.8)	138	253	
Female	567 (59.2)	174	393	
Region				0.07
Afghanistan	64 (6.7)	20	44	
Baluchistan	10 (1.1)	4	6	
Khyber Pakhtunkhwa	176 (18.4)	58	118	
Punjab	657 (68.6)	206	451	
Sindh	20 (2.1)	9	11	
Federally Administered Tribal Area	15 (1.6)	9	6	
Gilgit Baltistan	4 (0.4)	3	1	
Azad and Jammu Kashmir	11 (1.1)	3	8	
Cancer Type				0.001
Brain Cancer	6 (0.6)	6	0	
Breast Cancer	240 (25)	67	173	
Colorectal Cancer	98 (10.2)	33	65	
Gastrointestinal Cancer	12 (1.3)	8	4	
Head & Neck	192 (20)	68	124	
Hepatocellular Carcinoma	9 (1)	1	8	
Lung Cancer	27 (2.8)	5	22	
Lymphoma	1 (0.1)	1	0	
Ovarian Cancer	52 (5.4)	19	33	
Endometrial Cancer	13 (1.4)	8	5	
Cervical Cancer	3 (0.3)	1	2	
Pancreatic Cancer	35 (3.6)	17	18	
Renal Cancer	143 (15)	43	100	
Testicular Cancer	21 (2.2)	7	14	
Bladder Cancer	1 (0.1)	1	0	
Sarcoma	13 (1.4)	5	8	
Thyroid Cancer	92 (9.6)	22	70	

**Figure 3:**
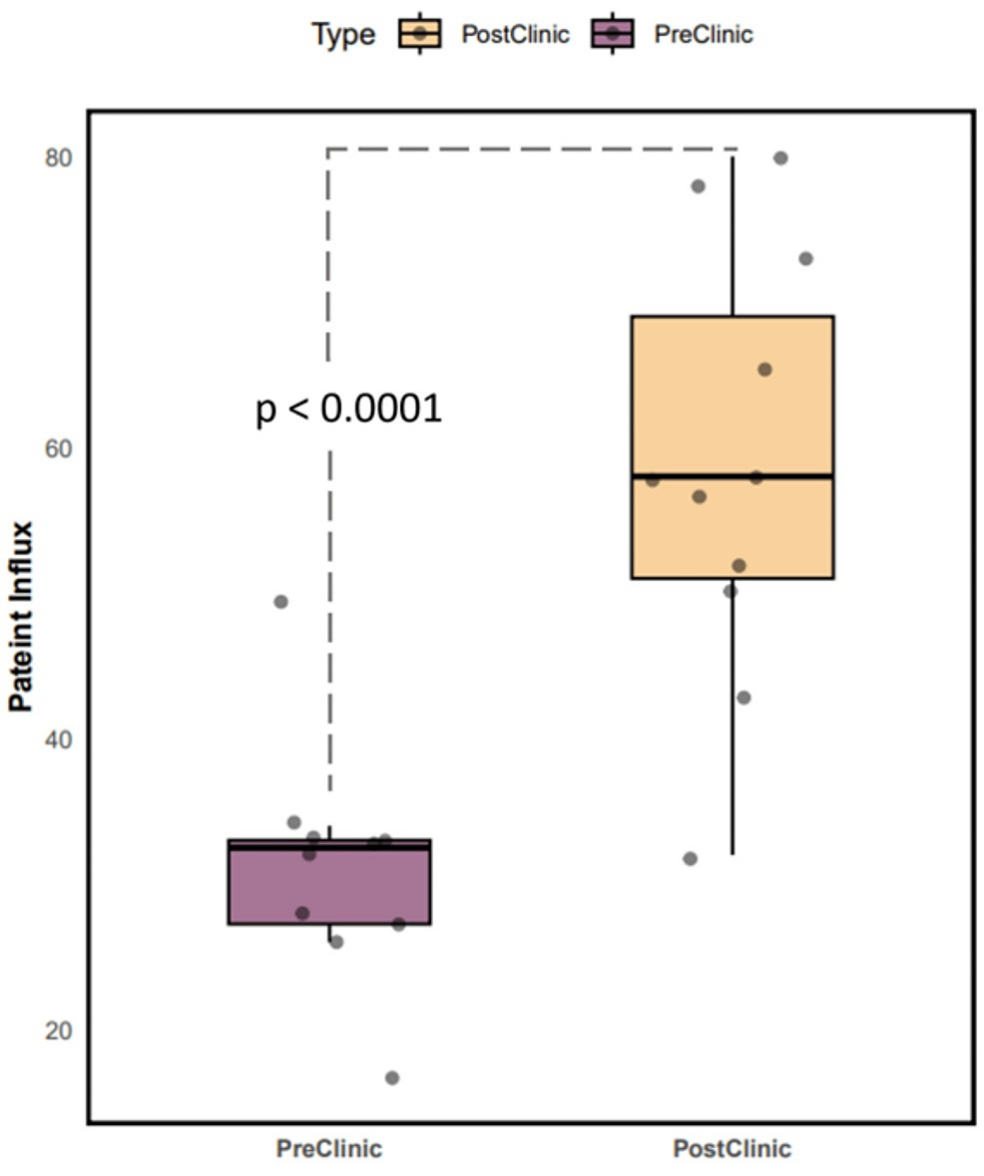
Patterns of cancer patient engagement in research post-biobank clinic. An independent t-test was performed to analyze the difference in the patient influx (average number of participants) enrolled in biobank research pre-clinic and post-clinic.

Before the introduction of the biobank clinic, we had secured participation from 312 patients. However, with the establishment of this clinic, we witnessed a significant difference in patient enrollment (p-value<0.001) as illustrated in Figure 3. Over an 11-month timeframe, we scheduled appointments for 759 patients. Notably, 656 patients actively visited the clinic, resulting in the enrollment of 646 patients. This achievement is underscored by an exceptional consent rate of 98.47%, affirming the strong commitment of these individuals to participate in our research initiatives. Among the 759 patients initially scheduled, 103 patients did not visit the clinic, and 10 individuals declined participation in the research.

## Discussion

The landscape of cancer research is advancing rapidly, with a growing focus on personalized therapy [10-13]. This cutting-edge strategy is designed to customize cancer treatments according to each patient's unique genetic and molecular characteristics, a move that promises to enhance treatment effectiveness while minimizing side effects [14-17]. This evolving research landscape holds immense potential for improving patient outcomes and reshaping the future of cancer treatment. However, Pakistan faces a multitude of challenges that significantly hinder its healthcare and research capabilities. These challenges are deeply rooted in a complex interplay of socioeconomic factors and systemic limitations. To unlock the benefits of personalized therapy and propel cancer research forward, it is imperative to establish a diverse repository of cancer patient samples and data within Pakistan. This invaluable resource will enable the country to leverage the latest advances in cancer treatment, resulting in more precise, effective therapies and improved patient outcomes.

Following our research objectives, SKMCH&RC has established a biobank and integrated a biobank research clinic within the hospital environment. The substantial connectivity between our biobank, clinic, and the electronic medical information of patients stands out as a notable strength. This deliberate integration draws inspiration from the successful framework of the Mayo Clinic Biobank, which capitalizes on its position within the Mayo Clinic healthcare system [18]. Utilizing this strategic location, the Mayo Clinic Biobank adeptly accesses electronic health record (EHR) data to passively gather follow-up information [18], a model that serves as both an inspiration and a benchmark for our biobank clinic, where we aspire to replicate and refine this efficient approach.

These initiatives are designed to elevate awareness about cancer research and actively engage the cancer patient community in research endeavors. We observed a significant rise in patient involvement, indicating a growing interest and willingness among cancer patients to actively engage in research initiatives. This surge in patient interest aligns with the findings of a study conducted by Lee et al. in which a substantial number of women enrolled in a breast cancer screening registry expressed their willingness to contribute to the biobank as well [19]. Additionally, we noted a significant increase in post-clinic patient numbers, underscoring the expanding reach and impact of the biobank clinic. 

This growth in patient engagement demonstrates the clinic's effectiveness in attracting individuals at the early stages of their cancer journey. Through these collective efforts, Pakistan can make significant strides in the realm of cancer research, offering renewed hope and improved prospects for those affected by this complex disease.

Although we extensively searched for previously published clinic-based biobank studies, we did not come across any relevant findings, indicating that our investigation represents the first clinic-based biobank research initiative undertaken in Pakistan. The absence of prior studies in this domain underscores the critical need for initiatives that bridge the gap in our understanding of the intersection between clinic-based biobanking and medical research in the Pakistani context. This novel undertaking not only fills a significant space in the current body of knowledge but also lays the groundwork for future investigations and advancements in healthcare practices within the country.

The current study has a few limitations. Currently, the eligible population for participation in the biobank is limited to treatment-naïve patients scheduled for upfront surgeries, resulting in a relatively small pool of eligible candidates. Additionally, the study is conducted at a single center due to the prevailing constraints in research infrastructure and standardized practices across the country. Nonetheless, our efforts extend to encompass patients from diverse regions and socioeconomic backgrounds in Pakistan. Within the existing resource constraints, facilities like this can significantly contribute to the advancement of patient-oriented research.

## Conclusions

The establishment of the biobank clinic marked a significant milestone in our mission to enhance cancer research awareness and participation within the cancer patient community. By fostering collaboration and participation, SKMCH&RC aims to bridge the gap between research and patient care, ultimately paving the way for more targeted and effective therapeutic interventions.
